# The *Fusarium sacchari* Effector FsP4H1 Targets the Transcription Factor DPb to Disrupt Catalase‐Mediated H_2_O_2_ Homeostasis and Suppress Host Immunity

**DOI:** 10.1111/mpp.70279

**Published:** 2026-05-19

**Authors:** Qianqian Ren, Chunping Cen, Huifang Li, Shichao Wang, Yixue Bao, Muqing Zhang, Wei Yao, Qin Hu

**Affiliations:** ^1^ College of Agriculture, Guangxi Sugarcane Bio‐Breeding Laboratory, State Key Laboratory of Conservation and Utilization of Subtropical Agro‐Bioresources, Guangxi Key Laboratory of Sugarcane Biology Guangxi University Nanning China

**Keywords:** effector, *Fusarium sacchari*, plant immunity, sugarcane, transcription factor

## Abstract

Pokkah boeng disease has been reported in nearly all countries where sugarcane is commercially cultivated. Historically, this disease was regarded as a minor concern; however, due to the impacts of climate change, it has now escalated into a significant issue. The causative agents of this disease are fungi, specifically belonging to the *Fusarium* fungal complex, with *Fusarium sacchari* being the predominant species in Guangxi Zhuang Autonomous Region, China. However, the pathogenic mechanism remains largely unknown. In this study, we have identified and characterized an effector protein designated as FsP4H1, which contains a prolyl‐4‐hydroxylase domain and is crucial for the virulence of *F. sacchari*. FsP4H1 localizes to the host cytoplasm and nucleus, and its nuclear localization is required for the immune suppression in the host. We demonstrate that FsP4H1 interacts with the transcription factor DPb, a positive regulator of plant immunity, and facilitates degradation of DPb via the 26S proteasome. This degradation leads to downregulation of catalase genes, *ScCAT9*, *ScCAT34* or *NbCAT*, which are direct transcriptional targets of DPb and play a crucial role in maintaining hydrogen peroxide (H_2_O_2_) homeostasis. Consequently, in the interaction between *F. sacchari* and sugarcane, *F. sacchari* secretes FsP4H1 into the hosts' nuclei, resulting in the disruption of the DPb–CAT module to enhance host susceptibility. Our study reveals a novel virulence strategy in which a fungal effector hijacks a conserved plant transcriptional network to suppress antioxidant defences, providing potential targets for resistance breeding in sugarcane.

## Graphical abstract

*FsP4H1* interacts with the transcription factor *DPb*, a positive regulator of plant immunity, and facilitates its degradation via the 26S proteasome. This degradation leads to downregulation of catalase genes *ScCAT9*, *ScCAT34* or *NbCAT* to enhance host susceptibility.

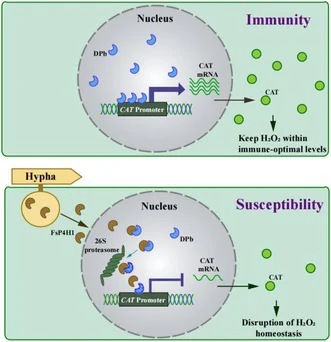

## 1 Introduction

Sugarcane is a vital global crop for sugar and bioenergy (Poorniammal et al. 2024), yet its production is severely threatened by fungal diseases. Among these, pokkah boeng disease (PBD), caused by the *Fusarium fujikuroi* species complex (FFSC), is increasingly destructive (Bao et al. 2023). Key pathogenic species such as *Fusarium sacchari* and *Fusarium verticillioides* induce symptoms ranging from leaf chlorosis to lethal top rot, causing PBD (Xu et al. 2019; Bao et al. 2023). The disease management has relied heavily on chemical fungicides like carbendazim, but their efficacy is diminishing due to emerging resistance in FFSC populations, posing a significant challenge (Xu et al. 2019). Fungal effector proteins are key weapons for pathogens to infect plants. They promote susceptibility by interfering with plant immune receptors, inhibiting the activity of defence‐related enzymes, and manipulating hormone signalling pathways (Lopez et al. 2008; Pieterse et al. 2009; Lo Presti et al. 2015). Therefore, elucidating the interaction between *F. sacchari* effectors and sugarcane is critical for improving disease resistance, but current research in this area remains limited.

Prolyl‐4‐hydroxylase (P4H), a member of the Fe(II)/α‐ketoglutarate‐dependent dioxygenase superfamily, catalyses the most prevalent post‐translational modification in biology: the hydroxylation of proline (Gorres and Raines 2010). This enzyme is widely distributed across animals, plants, microorganisms, and viruses, regulating diverse biological processes by generating 4‐hydroxyproline (Hyp) (Myllyharju 2003; Gorres and Raines 2010; Schnicker and Dey 2016). In animals, P4Hs are primarily divided into collagen‐type P4Hs (C‐P4Hs), which play a central role in collagen biosynthesis, and hypoxia‐inducible factor P4Hs (HIF‐P4Hs), which primarily regulate the hypoxic stress response(Schnicker and Dey 2016). In plants, P4H proteins are crucial for development and stress responses. Overexpression of the *Arabidopsis* *AtP4H1* protein induces a hypoxia‐like phenotype (Asif et al. 2009). Although the physiological role of P4H has been partially elucidated in vertebrate and plant models, its functional roles in fungi remain largely uncharacterized.

The E2F/DP transcription factor family plays a pivotal role in regulating cell cycle progression, DNA replication, and cell differentiation in both plants and animals (Lam and La Thangue 1994; Magyar et al. 2000). In plants, E2F and DP proteins form heterodimers that control the expression of genes required for the G1/S transition and S‐phase entry (Helin et al. 1993; Magyar et al. 2000). The typical E2Fs (E2Fa, E2Fb, E2Fc) require dimerization with DP proteins (DPa or DPb) for efficient DNA binding and transcriptional activity (Sekine et al. 1999; Mariconti et al. 2002; Perrotta et al. 2021). These complexes are negatively regulated by the retinoblastoma‐related (RBR) protein, which binds to E2F‐DP heterodimers and represses their transcriptional activity (Del Pozo et al. 2007). In addition to their classic roles in cell proliferation, recent studies have revealed that E2F‐DP complexes also regulate secondary cell wall biosynthesis and fibre development in woody plants (Wang, Quan, et al. 2024). Furthermore, allelic variations in DP genes can influence protein–protein interactions and transcriptional efficiency, thereby affecting plant growth and biomass traits (Wang, Quan, et al. 2024). This highlights the functional diversity and regulatory complexity of the E2F‐DP module in plant biology. Nevertheless, most research on the DP protein has focused on its role when binding to E2F, while the independent function of DP protein remains to be further elucidated.

In plants, catalases (CATs) are key enzymes that help remove hydrogen peroxide (H_2_O_2_), a harmful byproduct generated during photorespiration and in response to pathogen attack. By controlling H_2_O_2_ levels, CATs play important roles in both plant development and immune responses (Amna et al. 2010; Mhamdi et al. 2018). Early studies showed that *Arabidopsis* produces three different CAT enzymes, each exhibiting activity during different time periods. Among them, CAT2 is the most active in photosynthetic tissues and is quickly activated within minutes after plants detect bacterial infection signals, suggesting it helps trigger basic immune defences (Vandenabeele et al. 2010; Zimmermann et al. 2010). Subsequent studies revealed that two *Phytophthora sojae* effectors associate with catalase, thereby inhibiting host programmed cell death (Zhang et al. 2015). Recent studies have shown that the expression of catalase is finely regulated by multiple transcription factors, affecting the susceptibility of plants to pathogens (Riseh et al. 2024). Here, we report the effector protein FsP4H1, which contains an N‐terminal secretory signal peptide. FsP4H1 localizes to the cytoplasm and nucleus of plant cells, with its virulence dependent on nuclear localization. Knockout of *FsP4H1* reduced its pathogenicity toward sugarcane. We found that FsP4H1 interacts with the transcription factor ScDPb and induces degradation of ScDPb by the 26S proteasome. Consequently, FsP4H1 suppresses the transcription of *ScCAT*, a gene activated by ScDPb, leading to enhanced host susceptibility. This study elucidates a molecular mechanism by which *F. sacchari* uses a nuclear‐localized effector to disrupt the host transcriptional network, providing a potential target for sugarcane disease‐resistant breeding.

## 2 Results

### 2.1 Analysis of *FsP4H1*

**FIGURE 1:**
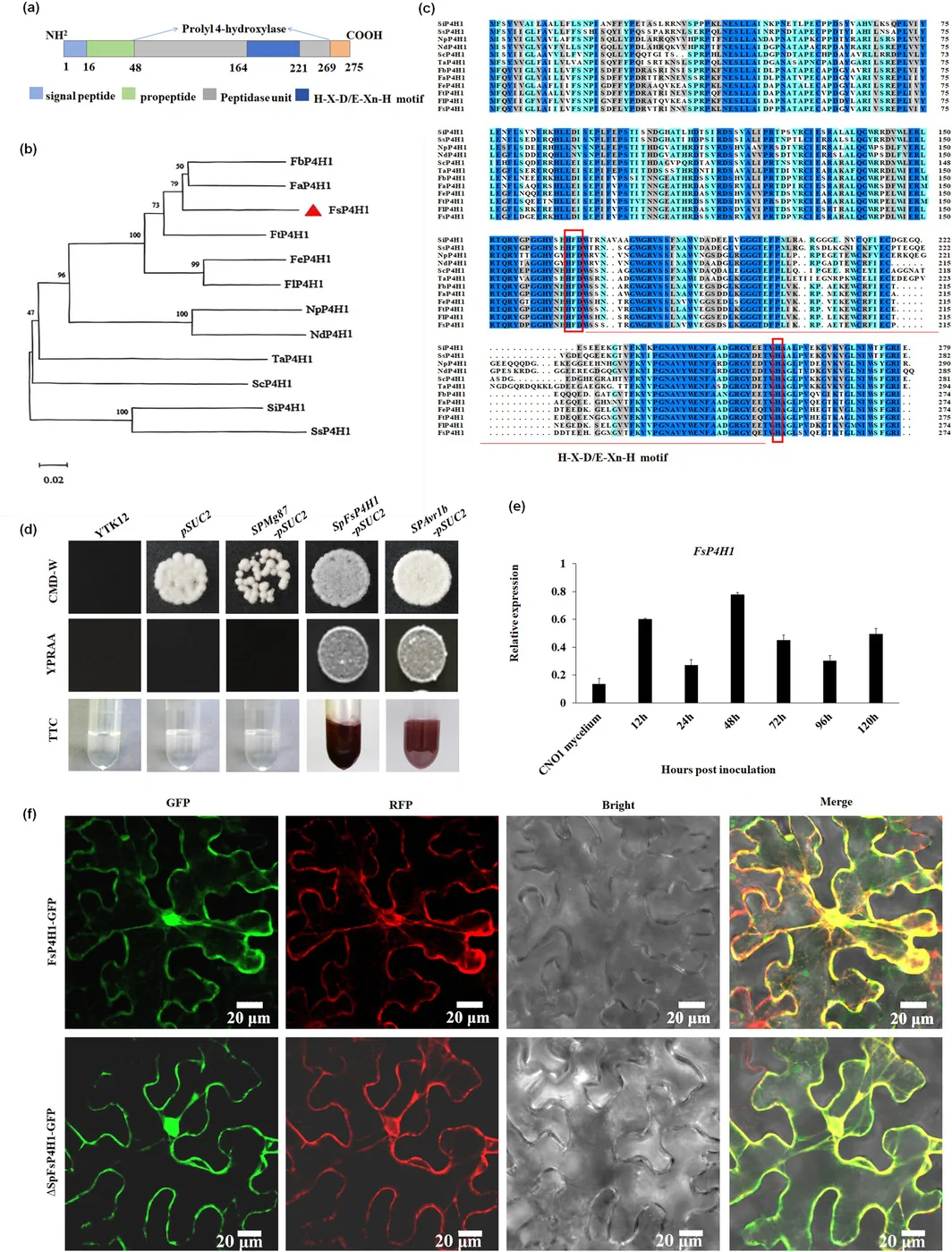
Characterization of FsP4H1. (a) Schematic view of FsP4H1 protein domains. (b) Phylogenetic analysis of FsP4H1 and prolyl 4‐hydroxylase from other fungi. The tree was constructed using the neighbour‐joining algorithm. (c) Multiple sequence alignment of FsP4H1 and its homologues. (d) Determination of the secretory function of the predicted signal peptide of FsP4H1 by a yeast secretion system and an invertase activity assay. (e) Expression profile of FsP4H1 during *Fusarium sacchari* infection of sugarcane. Data are means ± SD, *n* = 3. (f) Subcellular localization analysis of FsP4H1 and ΔSpFsP4H1 in *Nicotiana benthamiana* leaves. GFP or RFP fluorescence of FsP4H1‐GFP, ΔSpFsP4H1‐GFP and AtPLC2‐RFP (plasma membrane localization marker) were observed in the epidermal cells using confocal microscope. Scale bar = 20 μm.

Based on the previous identification of effector proteins of *F. sacchari* in sugarcane, a cDNA fragment of 825 bp containing a prolyl 4‐hydroxylase domain was amplified from the cDNA of *F. sacchari* and designated as *FsP4H1*. *FsP4H1* encodes a 275‐amino acid protein with a 16‐amino acid N‐terminal signal peptide and a typical iron‐binding motif H‐X‐D/E‐Xn‐H (Figure 1a). A phylogenetic tree was constructed using the the neighbour‐joining method to trace the evolutionary relationships between FsP4H1 and P4H proteins from other fungal species. As depicted in the constructed evolutionary tree, homologues of FsP4H1 are widely distributed among plant‐pathogenic fungi, and FsP4H1 was closely related P4H proteins from *Fusarium* species, including *Fusarium austroafricanum*, *Fusarium beomiforme* (Figure 1b). Multiple sequence alignment demonstrated substantial conservation of P4H proteins among diverse *Fusarium* species (Figure 1c).

### 2.2 Secretory Protein FsP4H1 Plays an Essential Role During Infection and is Localized in the Host Cytoplasm and Nucleus

To assess the secretory function of the FsP4H1 signal peptide, we performed a yeast secretion trap assay. The signal peptide directed fusion invertase secretion in the invertase‐deficient YTK12 strain, enabling growth on sucrose‐selective medium and catalysing the reduction of 2,3,5‐triphenyltetrazolium chloride (TTC) to the insoluble red‐coloured triphenylformazan, thereby confirming the secretory activity of the FsP4H1 signal peptide (Figure 1d). Reverse transcription‐quantitative PCR (RT‐qPCR) was used to investigate the expression of the *FsP4H1* in *F. sacchari* during its infection process in sugarcane leaves. It was found that *FsP4H1* expression peaked at 48 h post‐inoculation (hpi), reaching a level six times higher than that in uninvading host mycelium (Figure 1e), suggesting that *FsP4H1* may play a significant role in the infection by *F. sacchari*. Subsequently, we fused green fluorescent protein to the C‐terminal end of FsP4H1 (FsP4H1‐GFP), and the transient expression of FsP4H1‐GFP in *Nicotiana benthamiana* epidermal cells revealed dual localization in the nucleus and cytoplasm of FsP4H1 (FsP4H1‐GFP fluorescence did not entirely coincide with the RFP plasma membrane marker; notably, pronounced GFP fluorescence was also detectable in the cytoplasm). Furthermore, the localization remained unchanged after deletion of the signal peptide from FsP4H1‐GFP (∆SpFsP4H1‐GFP) (Figure 1f).

### 2.3 *FsP4H1* Is Required for the Full Virulence of *F. sacchari*

**FIGURE 2:**
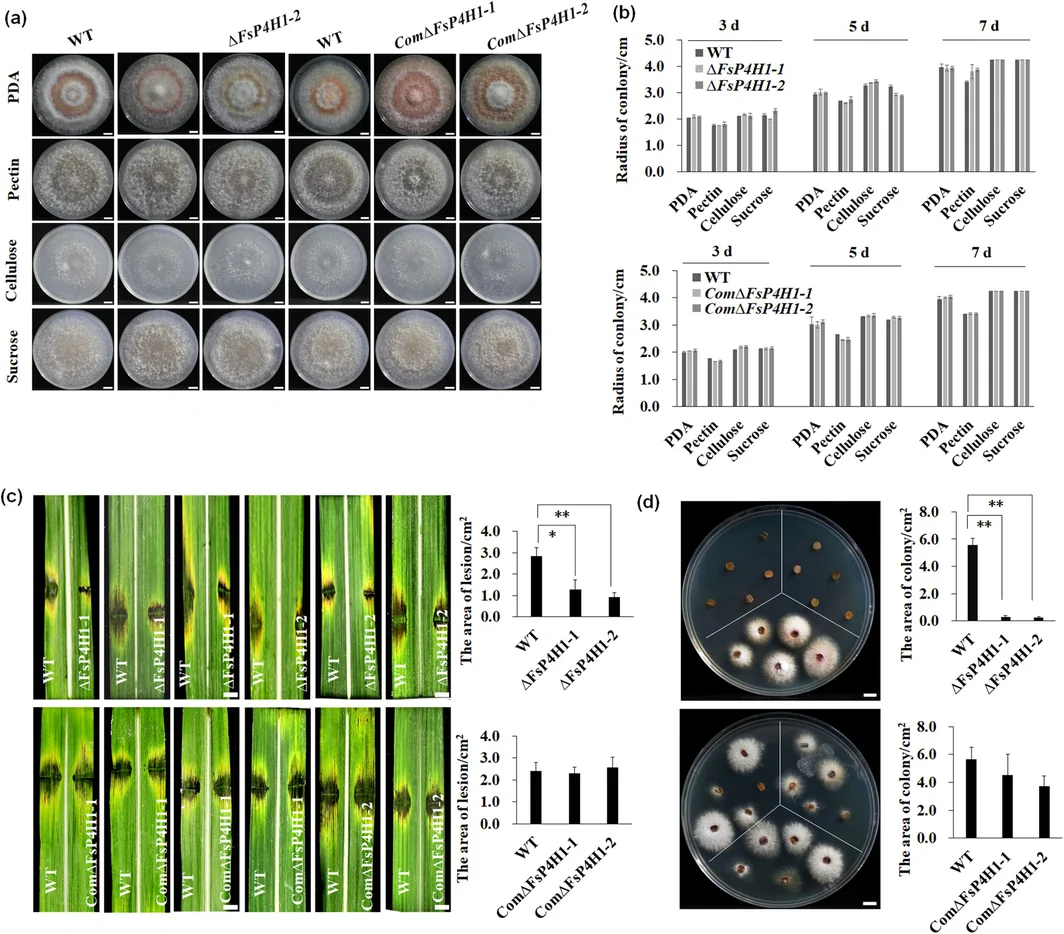
*FsP4H1* is dispensable for vegetative growth and conidiation of *Fusarium sacchari* but is required for full pathogenicity. (a) The growth phenotypes of Δ*FsP4H1*, *Com*Δ*FsP4H1* and wild‐type strains grown on potato dextrose agar (PDA) with pectin, cellulose or sucrose as carbon source for 7 days. (b) Statistical analysis of the vegetative growth rate of Δ*FsP4H1*, *Com*Δ*FsP4H1* and wild‐type strains grown on PDA with pectin, cellulose or sucrose as carbon source. Data are means ± SD, *n* = 6. (c) The pathogenicity of Δ*FsP4H1*, *Com*Δ*FsP4H1* and wild‐type (WT) strains on sugarcane leaves. Representative leaves were photographed at 7 days post‐inoculation. Data are means ± SD, *n* = 6; *p < 0.05, **p < 0.01 by Student's *t* test. (d) Recovery of Δ*FsP4H1*, *Com*Δ*FsP4H1* and wild‐type (WT) hyphae from infected leaves plated on PDA. Representative photographs were taken at 5 days. Data are means ± SD, *n* = 6; **p < 0.01 by Student's *t* test. Scale bar = 1 cm.

To further examine the role of *FsP4H1* in *F. sacchari* virulence, we created two *FsP4H1* knock‐out strains (Δ*FsP4H1‐1* and Δ*FsP4H1‐2*) and two complemented strains (*Com*Δ*FsP4H1‐1* and *Com*Δ*FsP4H1‐2*) using *Agrobacterium tumefaciens* ‐mediated *F. sacchari* transformation (Figure S1a,b). The *FsP4H1* knock‐out and complemented strains were verified by PCR and Southern blotting as shown in Figure S1c–e. To determine whether FsP4H1 is required for the normal growth and development of *F. sacchari*, we cultured the wild‐type strain CNO‐1, Δ*FsP4H1* and *Com*Δ*FsP4H1* in media with different carbon sources. The results showed no differences in growth rate, spore count, or spore morphology between CNO‐1 and the *FsP4H1* mutant strains, indicating that *FsP4H1* does not participate in the growth and development of *F. sacchari* (Figure 2a,b and Figure S2).

To investigate the contribution of FsP4H1 to virulence in the host plant, we inoculated sugarcane leaves with wild‐type (WT), Δ*FsP4H1* and *Com*Δ*FsP4H1* strains. At 120 h post‐inoculation (hpi), the Δ*FsP4H1* mutants exhibited significantly attenuated virulence, producing smaller lesion areas than that of WT (Figure 2c). The complemented strains *Com*Δ*FsP4H1* fully restored the pathogenicity, as the *Com*Δ*FsP4H1* strains generated lesions statistically comparable to those observed in the WT (Figure 2d). Simultaneously, fungal recovery assays confirmed that Δ*FsP4H1* mutants exhibited significantly reduced pathogen biomass compared to WT and ComΔFsP4H1 mutants. These findings demonstrate that *FsP4H1* is essential for the pathogenicity of *F. sacchari*.

### 2.4 *FsP4H1* Suppressed Host Immune Responses and Increased Susceptibility to *Botrytis cinerea*

**FIGURE 3:**
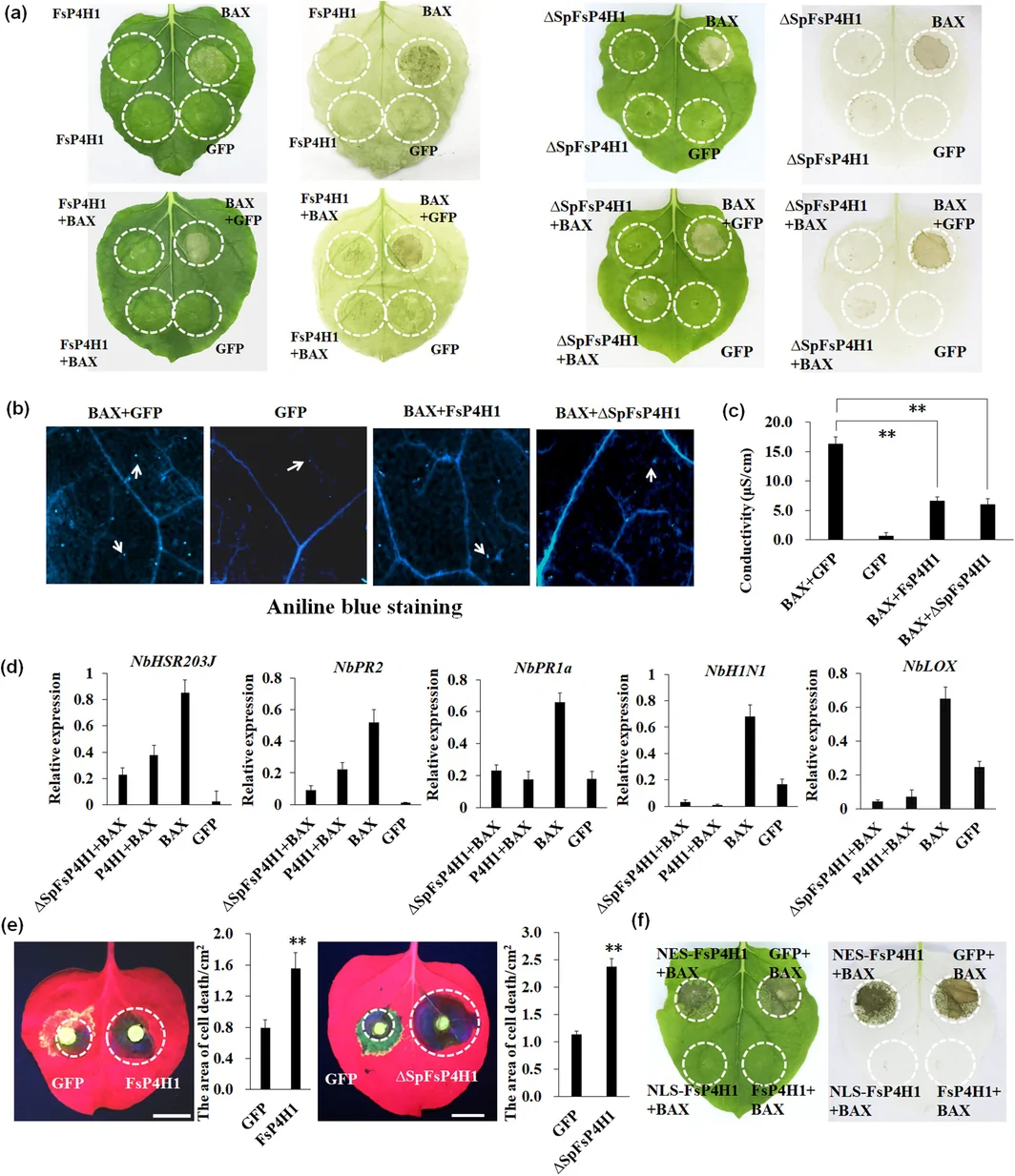
Functional characterization of FsP4H1 protein. (a) FsP4H1 suppressed BAX‐induced programmed cell death in *Nicotiana benthamiana*. GFP and BAX served as negative and positive control, respectively. Photos were taken 5 days after infiltration. The white circle represents the agroinfiltration site. (b, c) Callose deposition, as determined by aniline blue staining, in *N. benthamiana* leaves expressing FsP4H1. Data are means ± SD, *n* = 6; **p < 0.01 by Student's *t* test. (d) Relative expression of defence‐related genes in *N. benthamiana* leaves transiently expressing the indicated constructs. Data are means ± SD, *n* = 3. (e) Lesion diameters on *N. benthamiana* leaves expressing the indicated constructs after *Botrytis cinerea* inoculation; UV‐illuminated images at 2 days post‐inoculation. Lesion areas quantified at 2 days post‐ *B. cinerea* inoculation in *N. benthamiana* leaves transiently expressing the indicated constructs. Data are means ± SD, *n* = 6; **p < 0.01 by Student's *t* test. (f) *N. benthamiana* leaves were separately infiltrated with the indicated *Agrobacterium tumefaciens* suspensions to detect the function of nucleus‐localized FsP4H1 in manipulating plant immune responses. Photos were taken 5 days after infiltration.

Our previous discovery that *FsP4H1* plays a crucial role in the virulence of *F. sacchari* prompted us to further investigate the biological function of *FsP4H1* in plant defence. To investigate the role of *FsP4H1* in manipulating plant immune responses, we transiently expressed *FsP4H1* and its signal peptide‐deleted variant (Δ*SpFsP4H1*) in *N. benthamiana* leaves via *Agrobacterium*‐mediated infiltration. Neither *FsP4H1* nor Δ*SpFsP4H1* induced cell death, but both suppressed BAX (Bcl‐2‐associated X protein)‐induced cell death (Figure 3a). These results demonstrate that the FsP4H1 could suppress the host immune responses, and the signal peptide of FsP4H1 was dispensable for its cell death‐suppressive function.

Consistent with its cell death‐suppressive activity, co‐expression of *FsP4H1* or Δ*SpFsP4H1* with BAX attenuated callose deposition (Figure 3b,c) and downregulated defence‐associated genes (Figure 3d). To evaluate the impact on disease susceptibility, *N. benthamiana* leaves transiently expressing *FsP4H1* or Δ*SpFsP4H1* (with GFP as a negative control) were inoculated with *Botrytis cinerea* mycelial plugs at 24 h post‐agroinfiltration. By 2 days post‐inoculation (dpi), lesion diameters on leaves expressing *FsP4H1* or Δ*SpFsP4H1* were significantly larger than those on GFP‐expressing controls (Figure 3e). To determine whether the host immunity suppressive activity of FsP4H1 is dependent on the nuclear localization, we constructed N‐terminal fusions with a nuclear export signal (NES) or nuclear localization signal (NLS) to generate NES‐FsP4H1 and NLS‐FsP4H1, respectively. Subcellular localization analysis by confocal microscopy revealed that NES fusion abolished the nuclear localization of FsP4H1, whereas the NLS fusion promoted robust nuclear localization, although a small amount of protein was still detectable in the cytoplasm (Figure S3a). NES‐FsP4H1 lost the ability to suppress BAX‐induced cell death (Figure 3f), and the enhanced susceptibility phenotype to *B. cinerea* was also abrogated, with lesion sizes comparable to NLS‐FsP4H1 and GFP controls (Figure S3b,c). Collectively, these data establish that FsP4H1 could suppress plant immunity, and its virulence activity is contingent upon nuclear localization in the host.

### 2.5 FsP4H1 Interacts With DP Transcription Factor From Sugarcane and *N. benthamiana*

**FIGURE 4:**
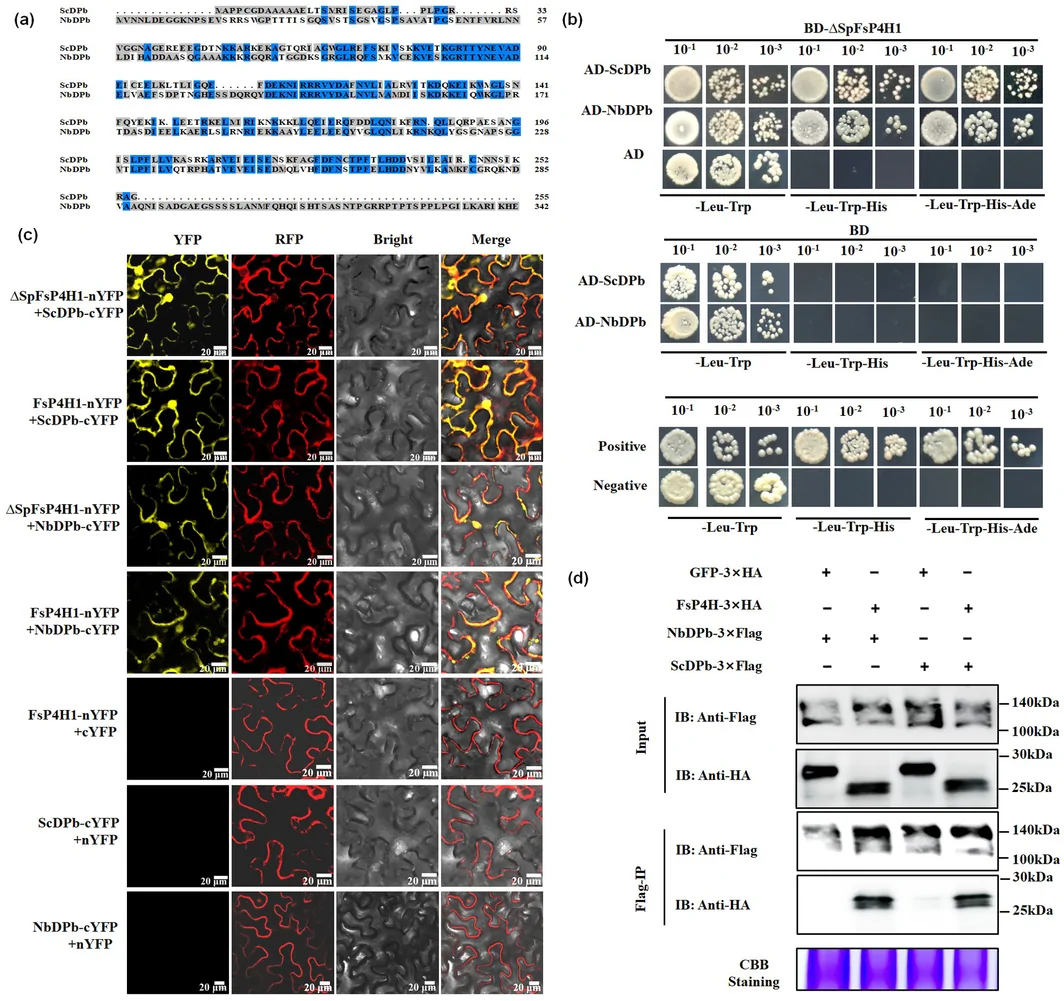
Direct interaction between ΔSpFsP4H1 and DP proteins ScDPb/NbDPb. (a) Multiple sequence alignment of ScDPb and its homologous protein NbDPb from *Nicotiana benthamiana*. (b) Yeast two‐hybrid assay showing interaction of ΔSpFsP4H1 with ScDPb and NbDPb. Δ*SpFsP4H1* was cloned into pGBKT7, *ScDPb* and *NbDPb* were cloned into pGADT7 vector. Δ*SpFsP4H1* (in pGBKT7‐BD) was co‐expressed with *ScDPb* or *NbDPb* (in pGADT7‐AD) and spotted on synthetic minimal medium. Growth on −Trp/−Leu/−His/−Ade (−TLHA) plates indicates interaction between the two proteins. (c) Bimolecular fluorescence complementation assays show that FsP4H1 and ΔSpFsP4H1 interact with ScDPb and NbDPb in planta. Reconstituted YFP fluorescence (yellow) was observed in both nucleus and cytoplasm after co‐expression of FsP4H1‐nYFP or ΔSpFsP4H1‐nYFP with ScDPb‐cYFP or NbDPb‐cYFP in *N. benthamiana* leaves. Scale bar = 20 μm. (d) Co‐immunoprecipitation assays showing that FsP4H1 interacts with DP proteins ScDPb/NbDPb. Total proteins were extracted from *N. benthamiana* leaves expressing with the indicated vectors. Immune complexes were pulled down using anti‐FLAG magnetic beads. DPb naturally heterodimerizes with E2F, thus the DPb‐3 × FLAG fusion protein migrates above 100 kDa, consistent with prior reports (Zhu et al. 2023).

To further elucidate the mechanism by which FsP4H1 mediates host susceptibility, a yeast two‐hybrid (Y2H) screen was performed using FsP4H1 as bait against a sugarcane cDNA library prepared from *F. sacchari*‐infected plants, and identified a potential interacting protein named ScDPb, which is a putative DPb transcription factor. The *N. benthamiana* homologue NbDPb (46% amino acid similarity with ScDPb) was subsequently cloned from leaf cDNA of *N. benthamiana* (Figure 4a). One‐to‐one Y2H assays also confirmed that FsP4H1 specifically interacted with both ScDPb and NbDPb in yeast (Figure 4b), and this result also indicates the conservation of the FsP4H1–DPb interaction in plants. Next, we performed bimolecular fluorescence complementation (BiFC) assays in *N. benthamiana* leaves. Before interaction analysis, confocal microscopy of individually expressed proteins confirmed that ScDPb and NbDPb localized to both the cytoplasm and nucleus (Figure S4c). For BiFC, FsP4H1 was fused to the N‐terminal YFP fragment, while ScDPb and NbDPb were each fused to the C‐terminal YFP fragment. Confocal imaging revealed that FsP4H1 interacted with both ScDPb and NbDPb, with YFP fluorescence detected in both nuclear and cytoplasmic compartments, indicating that these interactions occur in multiple subcellular locations in vivo (Figure 4c). Co‐immunoprecipitation (coIP) assays were also performed to confirm the FsP4H1–DPb interaction by co‐expressing FsP4H1 or GFP with ScDPb/NbDPb in *N. benthamiana* leaves. Total proteins were extracted from the co‐infiltrated leaves, and incubated with anti‐FLAG magnetic beads. Immunoblotting with anti‐HA antibody revealed ScDPb/NbDPb‐3 × FLAG specifically interacted with FsP4H1‐3 × HA, but not GFP‐3 × HA (Figure 4d).

### 2.6 DPb Enhances Plant Resistance and Is Degraded by FsP4H1

**FIGURE 5:**
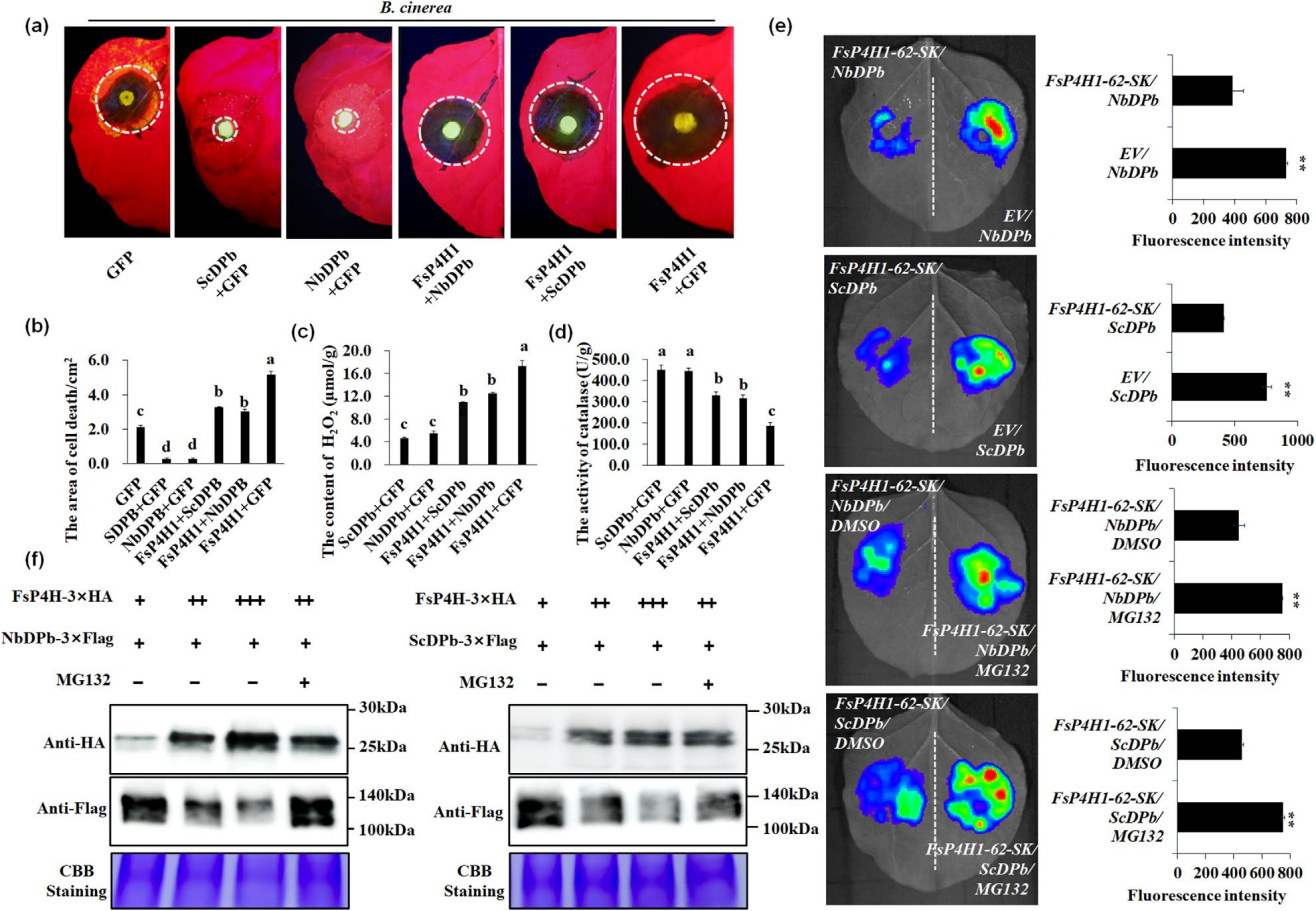
(a) Lesion diameters on *Nicotiana benthamiana* leaves expressing the indicated constructs after *Botrytis cinerea* inoculation, UV‐illuminated images at 2 days post‐inoculation. (b) Lesion areas quantified at 2 days post‐ *B. cinerea* inoculation in *N. benthamiana* leaves transiently expressing the indicated constructs. Data are means ± SD, *n* = 6. Different letters denote significant differences (one‐way ANOVA; *p* < 0.05). (c) H_2_O_2_ accumulation in *N. benthamiana* leaves transiently expressing the indicated constructs. Data are means ± SD, *n* = 6. Different letters denote significant differences (one‐way ANOVA; *p* < 0.05). (d) Catalase activity *in N. benthamiana* leaves transiently expressing the indicated constructs. Different letters denote significant differences (one‐way ANOVA; *p* < 0.05). (e) Luciferase assay showing that FsP4H1 promotes degradation of ScDPb‐LUC and NbDPb‐LUC in *N. benthamiana* at 48 h post‐infiltration; co‐infiltration with MG132 blocks this degradation, indicating the proteasome‐dependent degradation. Data are means ± SD, *n* = 3; **p < 0.01 by Student's *t* test. (f) Western blot showing that co‐expression of ScDPb/NbDPb with FsP4H1 reduced ScDPb/NbDPb abundance, and FsP4H1 affected the stability of ScDPb/NbDPb in a dose‐dependent manner. CBB, Coomassie Brilliant Blue staining of loading control.

To investigate the role of ScDPb/NbDPb in plant immunity, we performed transient co‐expression assays in *N. benthamiana*. ScDPb or NbDPb were co‐expressed with GFP or FsP4H1 for 24 h via agroinfiltration, using GFP alone and FsP4H1 with GFP as controls. Following inoculation with *B. cinere*a mycelial plugs, lesion areas were measured 2 dpi. Co‐expression of FsP4H1 and GFP resulted in the largest lesions, whereas co‐expression of ScDPb/NbDPb with GFP produced the smallest lesions (Figure 5a,b). Co‐expression of FsP4H1 with ScDPb/NbDPb resulted in intermediate lesion sizes, partially reducing the susceptibility caused by FsP4H1 (Figure 5a,b). Measurements of hydrogen peroxide (H_2_O_2_) accumulation and catalase activity were consistent with these disease phenotypes. ScDPb/NbDPb co‐expression with GFP maintained the lowest H_2_O_2_ level and the highest catalase activity, whereas FsP4H1 plus GFP accumulated the most H_2_O_2_ and exhibited the weakest catalase response (Figure 5c,d). Co‐expressing ScDPb/NbDPb with FsP4H1 yielded intermediate levels of both traits (Figure 5c,d). Collectively, these results indicate that ScDPb/NbDPb positively regulate disease resistance, likely by reducing H_2_O_2_ level, whereas FsP4H1 interferes with DPb function, resulting in excessive H_2_O_2_ accumulation.

To gain insights into the effect of FsP4H1 on ScDPb/NbDPb function, firefly luciferase (LUC) reporter assays were performed in *N. benthamiana*. FsP4H1, ScDPb‐LUC and NbDPb‐LUC were generated under the control of the 35S promoter, as indicated in Figure S5a. As shown in Figure 5e, equal concentrations and volumes of the indicated constructs were co‐infiltrated into *N. benthamiana* leaves by *A. tumefaciens* infiltration. The LUC (firefly luciferase) luminescence intensity, which indicates the protein level of recombinant ScDPb‐LUC or NbDPb‐LUC, decreased in the presence of FsP4H1, suggesting that FsP4H1 decreases the stability of ScDPb and NbDPb. To determine if the 26S proteasome mediates this degradation, infiltrated leaves were treated with the proteasome inhibitor MG132 or dimethyl sulphoxide (DMSO) (control), and the LUC activity of ScDPb‐LUC and NbDPb‐LUC in the *N. benthamiana* leaves was significantly restored upon treatment with MG132 (Figure 5e), demonstrating that FsP4H1 induces the degradation of ScDPb and NbDPb via the 26S proteasome pathway. Similarly, when we co‐expressed FsP4H1‐3× HA with ScDPb/NbDPb‐3× FLAG in *N. benthamiana* leaves, the results indicated that the degradation of ScDPb/NbDPb was increased with an increasing amount of FsP4H1, and the degradation of ScDPb/NbDPb was significantly inhibited by the treatment with MG132 (Figure 5f). Taken together, these results suggest that FsP4H1 decreases the stability of ScDPb/NbDPb, leading to hyperaccumulation of H_2_O_2_, thus resulting in the increased host susceptibility to pathogens.

### 2.7 DPb Functions as a Transcriptional Activator of CAT

**FIGURE 6:**
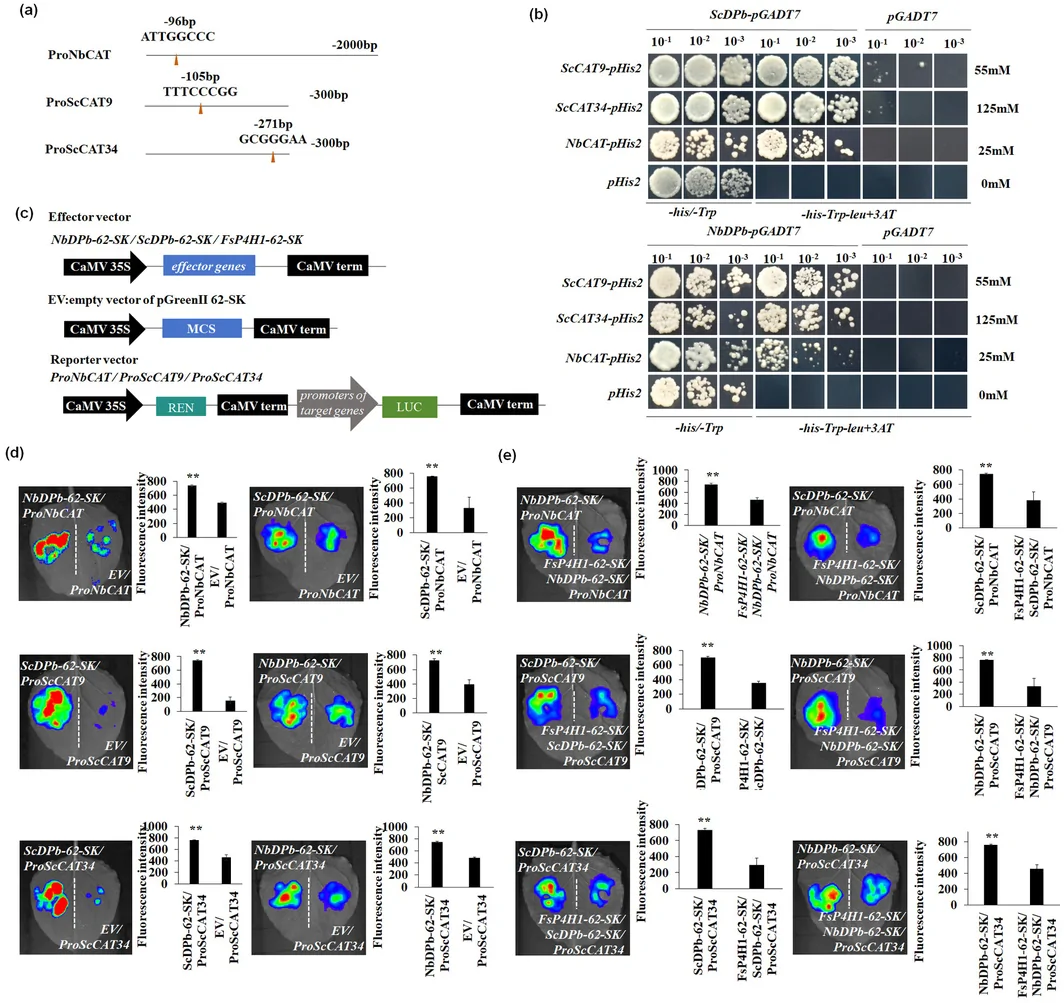
ScDPb directly activates ScCAT transcription and FsP4H1 represses this activation. (a) Diagram of DPb‐binding sites in the promoter regions of *ScCAT9*, *ScCAT34* and *NbCAT*. (b) Yeast one‐hybrid assay showing that ScDPb and NbDPb bind to the promoter fragments indicated in (a). Empty pGADT7 (AD) served as negative control. (c) Schematic representation of the luciferase (LUC) reporter and effector constructs used in transient assays. (d) LUC activity indicates the transcriptional level of catalase (CAT) promoters with or without ScDPb or NbDPb in *Nicotiana benthamiana* leaves. Data are means ± SD, *n* = 3; **p < 0.01 by Student's *t* test. (e) LUC activity indicates the transcriptional level of CAT promoters in *N. benthamiana* leaves; DPb‐activated LUC signals are markedly reduced upon co‐expression of FsP4H1, demonstrating that FsP4H1 suppresses DPb‐mediated transcriptional activation of CAT genes. Data are means ± SD, *n* = 3; **p < 0.01 by Student's *t* test.

Based on the significant differences in both H_2_O_2_ accumulation and catalase (CAT) activity among *N. benthamiana* expressing DPb alone, FsP4H1 alone, or co‐expressing DPb with FsP4H1, we hypothesised that CAT may constitute a direct regulatory target of DPb. Using *Arabidopsis thaliana* CAT1 (AT1G20630, TAIR10) as a query, we identified 12 *NbCAT* homologues in *N. benthamiana* and 34 *ScCAT* homologues in cultivated hybrid sugarcane ZZ1. As indicated in Figure 6a, promoter analysis revealed that one *N. benthamiana CAT* gene named *NbCAT1* (Nbe06g06210.1) and two sugarcane *CAT* genes designated as *ScCAT9* (ROC‐So‐Chr10B0027940) and *ScCAT34* (ROC‐Rec‐Chr01A0045830) contained the reported conserved DPb‐binding site previously identified in the human and plant systems (Zhu et al. 2023; Wang, Quan, et al. 2024). Yeast one‐hybrid (Y1H) assays demonstrated that ScDPb or NbDPb directly interacted with the promoters of *ScCAT9*, *ScCAT34* and *NbCAT* (Figure 6b). To functionally validate these interactions, we designed LUC reporter vectors (Figure 6c) and conducted luciferase assays in *N. benthamiana*. Co‐expression of ScDPb or NbDPb with promoters of *ScCAT9*, *ScCAT34* and *NbCAT*, significantly increased LUC activity compared to empty vector controls, confirming that both DPb proteins acted as transcriptional activators of their respective CAT targets (Figure 6d). Notably, when FsP4H1 was co‐expressed with these complexes, LUC activity was markedly reduced for all three *CAT* genes (Figure 6e), indicating that FsP4H1 suppresses DPb‐mediated transcriptional activation. Collectively, these results establish *ScCAT9*, *ScCAT34* and *NbCAT* as direct downstream targets of ScDPb and NbDPb and demonstrate that FsP4H1 interferes with this transcriptional regulation.

### 2.8 CAT Positively Regulates Plant Immunity

**FIGURE 7:**
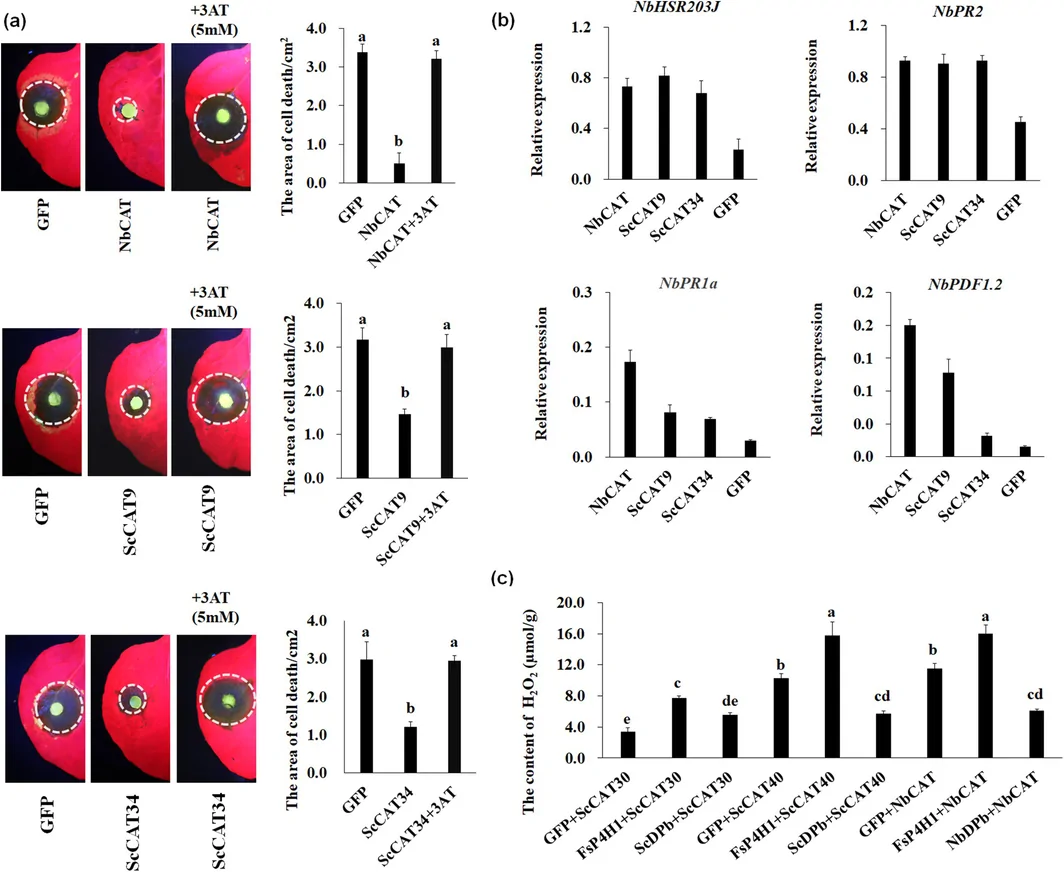
ScCAT9, ScCAT34 and NbCAT positively regulate plant immunity and are antagonized by FsP4H1. (a) Lesion diameters on *Nicotiana benthamiana* leaves expressing the indicated constructs after *Botrytis cinerea* inoculation; UV‐illuminated images at 2 days post‐inoculation. 5 mM 3‐AT was infiltrated where indicated. Lesion areas quantified at 2 days post‐ *B. cinerea* inoculation in *N. benthamiana* leaves transiently expressing the indicated constructs. Data are means ± SD, *n* = 6. Different letters denote significant differences (one‐way ANOVA; *p* < 0.05). (b) Relative expression of defence‐related genes in *N. benthamiana* leaves transiently expressing the indicated constructs. Data are means ± SD, *n* = 3. (c) H_2_O_2_ accumulation in *N. benthamiana* leaves transiently expressing the indicated constructs. Data are means ± SD, *n* = 6. Different letters denote significant differences (one‐way ANOVA; *p* < 0.05).

To determine whether ScCATs function as positive regulators of plant immunity, we transiently expressed *ScCAT9*, *ScCAT34*, *NbCAT* and *GFP* (control) in leaves of *N. benthamiana*. At 24 h post‐infiltration (hpi), the expression sites were inoculated with mycelial plugs of *B. cinerea* . In a separate control treatment, the catalase inhibitor 3‐amino‐1,2,4‐triazole (3‐AT) was co‐infiltrated at 24 hpi, with pathogen inoculation performed 12 h later. Lesion area measurements at 2 dpi demonstrated that expression of ScCAT9, ScCAT34 or NbCAT significantly reduced lesion size compared to the GFP control. However, 3‐AT treatment abolished this effect, resulting in lesion sizes comparable to the GFP control (Figure 7a). These findings indicate that ScCAT9, ScCAT34 and NbCAT positively regulate plant disease resistance.

Defence gene expression analysis further substantiated the immunomodulatory role of CAT. Transcript accumulation of defence‐associated genes such as *NbHSR203J*, *NbPR1a*, *NbPR2* and *NbPDF1.2* were significantly elevated in CAT‐expressing leaves relative to GFP controls (tissues harvested at 12 hpi from within 5 mm of the lesion margin) (Figure 7b). Co‐expression experiments revealed differential H_2_O_2_ accumulation, with CAT alone expression suppressing H_2_O_2_ levels, FsP4H1 co‐expression resulting in maximal H_2_O_2_ accumulation, and DPb co‐expression yielding intermediate values (Figure 7c). Together, these findings establish ScCATs as positive regulators of plant immunity and reveal that this activity is antagonized by FsP4H1.

## 3 Discussion

**FIGURE 8:**
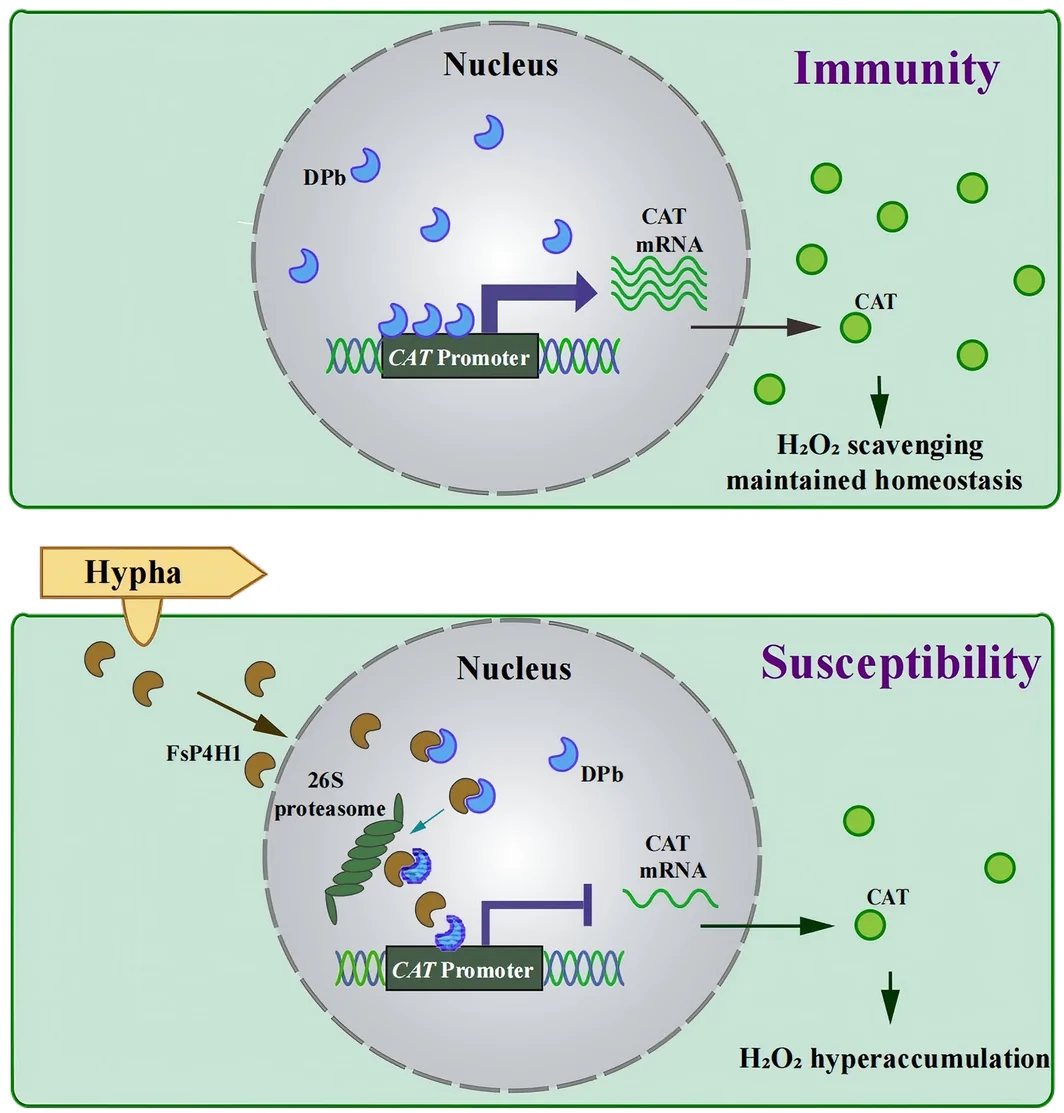
Proposed model of FsP4H1‐mediated suppression of plant immunity. FsP4H1 is secreted into the host nucleus where it interacts with the transcription factor ScDPb/NbDPb and promotes degradation of the transcription factor via the 26S proteasome pathway. ScDPb/NbDPb depletion abolishes the trans‐activation of *ScCAT9*, *ScCAT34* and *NbcCAT1*, leading to reduced catalase activity and sustained H_2_O_2_ accumulation that enhances host susceptibility to pathogen infection.

Fungal pathogens invade host plants by secreting multiple effector proteins that suppress immune responses and manipulate cellular processes (Giraldo and Valent 2013). In this study, we identified and characterized the secreted effector protein FsP4H1 from *F. sacchari*, which possesses a characteristic proline 4‐hydroxylase (P4H) domain, belonging to an evolutionarily highly conserved enzyme family. Our findings reveal that FsP4H1 enhances disease susceptibility by translocating into the host nucleus and mediating the proteasomal degradation of the sugarcane transcription factor ScDPB. This degradation leads to transcriptional repression of catalase genes, resulting in hydrogen peroxide (H_2_O_2_) hyperaccumulation (Figure 8). Notably, nuclear localization of FsP4H1 in host cells is a prerequisite for its suppression of the host immune response. During sugarcane infection by *F. sacchari*, the mechanism by which the fungal‐secreted effector FsP4H1 enters host cells and traffics to the nucleus, and whether this process depends on additional host or fungal auxiliary factors remains unclear. A comprehensive investigation of this trafficking pathway will significantly advance our understanding of the precise molecular mechanism through which FsP4H1 effectors contribute to virulence during the *F. sacchari*–sugarcane interaction.

Previous studies on P4H have primarily focused on vertebrate collagen biosynthesis or P4H isoenzymes in animals (Myllyharju 2003; Salo et al. 2024). In plants, P4H has been partially characterized from a few species, and one of the *Arabidopsis* P4Hs (*AtP4H1*) has been shown to hydroxylate proline‐rich peptides and participate in hypoxic stress responses (Asif et al. 2009). Notably, in this study, we demonstrate that FsP4H1 functions as a pathogen effector protein, evolving from a proline hydroxylase into a key negative regulator of immunity, dependent on its nuclear localization in the host (Figures 1, 2, 3).

DP protein is an indispensable partner for E2F transcriptional activity, primarily functioning through heterodimer formation with E2F (Kosugi and Ohashi 2002). Previous studies have demonstrated that DPb can generate multiple phosphorylated protein variants via alternative splicing, exhibiting tissue‐specific expression patterns and potentially diverse functional roles (Rogers et al. 1996). In this study, we identified ScDPb as an interacting partner of FsP4H1 and found that it positively regulates the host immune response against *F. sacchari* (Figure 4). Moreover, co‐expression assays further revealed that FsP4H1 targets ScDPb for degradation via the 26S proteasome pathway (Figure 5). This protein degradation pattern is similar to previous observations in mammalian systems, indicating that DPa which is not bound to E2F undergoes ubiquitin‐proteasome degradation (Magae et al. 1999). It not only indicates the evolutionary conservation of DP protein stability regulation across plants and animals, but also suggests that pathogens may exploit host cell‐cycle regulatory modules to attenuate plant immunity. Whether FsP4H1 triggers ScDPb degradation by disrupting the stability of the ScDPb–E2F complex remains an important question for future investigation.

Our results showed that ScDPb functions as a transcriptional activator by binding to the promoters of both *ScCAT9* and *ScCAT34*. Moreover, FsP4H1‐mediated degradation of ScDPb leads to a significant reduction in the expression of these two catalase genes (Figure 6). Plant CATs serve not only as antioxidant enzymes metabolizing H_2_O_2_, but also as regulatory switches within the immune network. They integrate pathogen signals, hormonal pathways, and environmental stresses by dynamically balancing reactive oxygen species (ROS) levels, demonstrating the remarkable functional diversity of plant CATs (Mhamdi et al. 2012; Baker et al. 2023; Riseh et al. 2024). In this study, ScCAT9, ScCAT34 and NbCAT function as positive regulators of plant immunity (Figure 7). Y1H and LUC assays further showed that either ScDPb or NbDPb directly activates the transcription of the *CAT* genes. *B. cinerea* inoculation revealed that elevated *CAT* expression restricts lesion expansion, while quantification of H_2_O_2_ accumulation indicates that CAT attenuates fungal ingress by scavenging excess H_2_O_2_ to maintain H_2_O_2_ homeostasis (Figures 6 and 7). Notably, the effector FsP4H1 promotes DPb degradation, thereby repressing CAT expression, disrupting H_2_O_2_ homeostasis, and facilitating fungal infection. Interestingly, this FsP4H1–ScDPb–ScCAT regulatory network exhibits high similarity to the recently described PsAvh113–GmDPB–GmCAT1 module in *P. sojae* (Zhu et al. 2023). In both cases, secreted effector proteins degrade host DPb transcription factors via the 26S proteasome, thereby suppressing catalase expression and impairing H_2_O_2_ detoxification, ultimately enhancing pathogen infection capacity. Despite the distant phylogenetic relationship between *F. sacchari* and *P. sojae*, they have convergently evolved similar strategies targeting DPb to inhibit CAT, suggesting DPb may represent a conserved immune regulatory node in plants. Similar regulation of catalase has been observed in other pathogenic systems, although the molecular mechanisms involved differ. The barley powdery mildew effector CSEP0027 disrupts H_2_O_2_ clearance mechanisms and promotes fungal colonization by mislocalizing the HvCAT1 protein (Yuan et al. 2021), whereas the wheat stripe rust effector Pst9653 disturbs the subcellular distribution of TaCAT3 to suppress H_2_O_2_ accumulation and facilitate infection (Wei et al. 2025). Notably, HvCAT1 and TaCAT3 regulate H_2_O_2_ levels through subcellular relocalization, suggesting the potential existence of analogous CAT in sugarcane, which may influence disease resistance by adjusting subcellular localization. This hypothesis warrants further investigation. These findings indicate that pathogens can interfere with catalase activity at either the transcriptional or post‐translational level. For example, *P. sojae* CRN effectors PsCRN63 and PsCRN115 differentially alter catalase stability and localization: PsCRN63 destabilizes the enzyme, elevates H_2_O_2_ levels, and reduces resistance, whereas PsCRN115 protects catalase, lowers H_2_O_2_ levels and similarly diminishes resistance (Zhang et al. 2015). This demonstrates that pathogen effectors in different systems have evolved diverse strategies to manipulate host catalase activity and H_2_O_2_ homeostasis to promote infection.

In summary, it can be inferred that the *Fusarium* effector protein FsP4H1 promotes the degradation of the host transcription factor ScDPb by binding to it, thereby inhibiting *ScCAT* expression. This results in a disruption of H_2_O_2_ homeostasis, thereby enhancing susceptibility to infection by pathogens. Taken together, the findings of this study suggest that the DPb‐CAT may constitute a potential defence mechanism, which is likely to be exploited by the effector from pathogen to advance infection.

## 4 Experimental Procedures

### 4.1 Plant Materials and Microbe Cultivation

*Nicotiana benthamiana* was cultured in a light chamber (28°C, 14‐h photoperiod, relative humidity 65% ± 5%). *F. sacchari*‐susceptible sugarcane cultivar Zhongzhe No. 1 (ZZ1) was grown in a greenhouse (28°C, 14‐h photoperiod, relative humidity 65% ± 5%). The *F. sacchari* isolate was grown on potato dextrose agar (Solarbio). *F. sacchari* knockout and complementation mutants were cultured on PDA supplemented with appropriate antibiotics. *B. cinerea* B05.10 was cultured on PDA at 23°C for disease resistance analysis. *Escherichia coli* DH5α was cultured at 37°C. *A. tumefaciens* GV3101 and AGL1 were cultured at 28°C for *Agrobacterium*‐mediated transformation of *N. benthamiana* and fungal transformation, respectively.

### 4.2 Bioinformatics Analysis

The sequence was submitted to InterPro (https://www.ebi.ac.uk/interpro/search/sequence/) to identify conserved functional domains. Protein signal peptides (SP) were predicted using SignalP‐6.0 (https://services.healthtech.dtu.dk/services/SignalP‐6.0/). The phylogenetic tree was constructed using MEGA 11.0 software with the neighbour‐joining algorithm. Amino acid sequence alignment was performed using DNAMAN software.

### 4.3 RNA Extraction and RT‐qPCR Assays

Total RNA was extracted from sugarcane leaves inoculated with *F. sacchari* at different time points using the Eastep Super Total RNA Extraction Kit (Promega). Reverse transcription was performed using the HiScript II Reverse Transcriptase Kit (Vazyme). Quantitative real‐time PCR qPCR was performed using a LightCycler 96 system (Roche). Sugarcane *GADPH* served as the reference gene for sugarcane, while *NbEF1* acted as the reference gene for *N. benthamiana*. Relative expression levels were determined using the 2^−ΔΔ*C*t^ method based on three independent biological replicates. All primers for RT‐qPCR are listed in Table S1.

### 4.4 Yeast Secretion Trap Assay

The predicted signal peptide of FsP4H1 was functionally validated using the yeast secretion trap assay, as described by Yin et al. (2018). The signal peptide DNA of FsP4H1 was cloned into the pSUC2 vector lacking a signal peptide, generating *SpFsP4H1‐pSUC2*. The empty vector pSUC2 and *SPMg87‐pSUC2* served as negative controls, while *SPAvr1b‐pSUC2* was used as a positive control. All constructs were transformed into the yeast strain YTK12, and the resulting transformants were initially screened on CMD−W (without tryptophan) medium. Positive clones were then transferred to YPRAA plates, and their secretory activity was assessed using the 2,3,5‐triphenyl tetrazolium chloride (TTC) reduction assay. Functional secretion reduces TTC to an insoluble red formazan.

### 4.5 Subcellular Localization Assays

The full‐length coding sequences of *FsP4H1*, Δ*SpFsP4H1* (a signal peptide deletion mutant), *ScDPb* and *NbDPb* were amplified using gene‐specific primer pairs (Table S1) and inserted into the BamHI*‐*KpnI‐digested pBI121‐GFP vector to generate C‐terminal GFP fusions driven by the CaMV 35S promoter. To obtain nuclear export constructs, the CDS of *FsP4H1* and Δ*SpFsP4H1* were cloned into the BamHI*‐*KpnI‐digested pCambia1300‐NES‐GFP vector, yielding N‐terminal NES (nuclear export signal) and C‐terminal GFP fusions. The *35S::AtPLC2‐RFP* plasmid served as a plasma membrane localization marker. All constructs were introduced into *A. tumefaciens* GV3101 by freeze–thaw transformation. *Agrobacterium* suspensions (OD₆₀₀ = 0.8) were infiltrated into 4‐ to 5‐week‐old *N. benthamiana* plants. The epidermal cells of *N. benthamiana* leaves were imaged 60 h post‐infiltration using an Olympus FV3000 confocal laser scanning microscope.

### 4.6 Construction of Knockout and Complementation Mutants

The generation of knockout and complementation mutants was conducted through the employment of an *Agrobacterium*‐mediated homologous recombination knockout and complementation strategy. Approximately 1 kb of sequence upstream and downstream of the *FsP4H1* gene was amplified from *F. sacchari* DNA. This single fragment was inserted into the PacI site of the hygromycin B‐resistant vector pGKO‐Hyg using the ClonExpress Ultra One Step Cloning Kit V2 (Vazyme) for constructing the knockout mutant. The construction of the complementary mutant was conducted through the amplification of a DNA fragment containing approximately 1 kb of the upstream region, the *FsP4H1* sequence, and approximately 1 kb of the downstream region. This fragment was inserted into the G418‐resistant pDHtsk vector at the HindIII/BamHI sites to generate the *FsP4H1* complementation construct. The recombinant plasmid was verified by PCR using the corresponding primers (Table S1). The validated recombinant plasmid was then transformed into *Agrobacterium tumefaciens* AGL1. According to the previously described method (Liu et al. 2024), the knockout mutant *Agrobacterium* was co‐cultured with *F. sacchari* CNO‐1 to screen for the knockout mutant fungal strain (Δ*FsP4H1*), and the knockout mutant fungal strain was complemented to screen for the complemented mutant fungal strain (*Com∆FsP4H1*). Spore suspensions of CNO‐1, Δ*FsP4H1* and *Com∆FsP4H1* (each at a concentration of 1 × 10^5^ spores/mL) were inoculated at the center of different carbon source media for cultivation. Colony morphology and radial growth were recorded at 3, 5 and 7 days post‐inoculation (dpi). For conidial quantification, 10 mL of sterile distilled water was added to each plate, and the colony was washed off. The resulting suspension was filtered through sterile filter paper to remove mycelial debris. The conidial concentration was determined using a haemocytometer, and conidial morphology was observed under a light microscope.

### 4.7 Southern Blotting

To verify that the Δ*FsP4H1* transformants were single‐copy insertional mutations, genomic DNA was extracted and 25 μg of DNA was digested with BamHI. The *Hyg* probe was amplified from plasmid pGKO‐Hyg using the primer pair Hyg‐probe‐F/R (Table S1). Southern hybridization experiments were then performed with a digoxigenin DNA labelling and detection kit (11,585,614,910, Roche) following the manufacturer's instructions.

### 4.8 Pathogenicity Assay

Pathogenicity assays of the *FsP4H1* knockout and complemented mutants were conducted as previously described (Hong et al. 2024). Wild‐type CNO‐1, Δ*FsP4H1* and *Com∆FsP4H1* spore suspensions were inoculated onto PDA and cultured for 7 days. Agar plugs (5 mm diameter) colonized by each strain were inoculated onto leaves of six‐leaf‐stage seedlings. Lesion sizes were measured at 5 dpi. Subsequently, the infected leaves (0.5 mm wide square leaf pieces were taken about 2 cm away from the initial inoculation point) were surface sterilized and then cultivated on PDA.

### 4.9 *Agrobacterium*‐Mediated Transformation in *N. benthamiana*

The full‐length coding sequence of target genes, GFP (freen fluorescent protein) and BAX (Bcl‐2‐associated X protein), were PCR‐amplified and inserted into the ClaI‐NotI sites of the potato virus X (PVX) binary vector pGR107 with a C‐terminal 3×HA tag. All resulting constructs were introduced into *A. tumefaciens* GV3101 and transiently expressed in 4‐ to 5‐week‐old *N. benthamiana* leaves as previously described with minor modifications (Carlson et al. 2023). BAX and GFP were used as positive and negative controls, respectively. All primers used for cloning are listed in Table S1.

### 4.10 ROS Burst, Callose and Catalase Activity Assay

The callose deposition in *N. benthamiana* were detected using the aniline blue staining method as described previously (Li et al. 2024). The determination of H_2_O_2_ and catalase activity was carried out using the Micro Hydrogen Peroxide Assay Kit and Catalase (CAT) Activity Assay Kit (Solarbio), respectively, following the manufacturer's instructions.

### 4.11 *Botrytis cinerea* Inoculation Assay

*Nicotiana benthamiana* leaves transiently expressing the target proteins were excised 24 h after agroinfiltration, and their petioles were wrapped with sterile water‐moistened absorbent cotton. A 5 mm mycelial plug of *B. cinerea* was placed on the agro‐infiltrated area. The inoculated leaves were placed in plastic containers lined with moist filter paper and cultured for 2 days at 23°C under a photoperiod of 16 h light/8 h dark. Lesion development was monitored daily from 2 to 5 dpi and photographed under UV light to visualize necrotic areas. For catalase activity inhibition, 5 mM 3‐amino‐1,2,4‐triazole (3‐AT) was re‐infiltrated into the identical agro‐infiltrated areas at 24 h post‐infiltration, followed immediately by *B. cinerea* inoculation.

### 4.12 Yeast One‐/Two‐Hybrid Assays

Promoter fragments of *ScCAT9* (300 bp containing the TTTCCCGG cis element), *ScCAT34* (300 bp containing the GCGGGAA cis element), and *NbCAT* (2000 bp containing the ATTGGCCC cis element) were inserted into the pHis2 vector to generate bait vectors. The coding sequences of *ScDPb* and *NbDPb* were cloned into pGADT7 (AD) to produce prey vectors. Bait vectors and an empty pGADT7 vector were co‐transformed with the prey vectors into the Y187 yeast strain via the polyethylene glycol/litium acetate yeast transformation method. The transformed colonies were plated on SD/−Trp/−Leu selective medium. Positive clones were cultured overnight in SD/−Trp/−Leu liquid medium, and 5‐μL aliquots were spotted onto SD/−Trp/−Leu and SD/−Trp/−Leu/–His plates supplemented with 3‐aminotriazole (3‐AT) at concentrations ranging from 5 to 125 mM to assess interaction strength.

A cDNA library derived from sugarcane leaves was previously constructed in the yeast vector pGADT7 using Matchmaker Library Construction and Screening Kits. The coding sequence of Δ*SPFsP4H1* was amplified and cloned into pGBKT7 to generate the bait vector BD‐ΔSPFsP4H1 at the EcoRI‐BamHI sites and transformed into yeast strain Y2H Gold for library screening as previously described (Wang, Wu, et al. 2024). We performed 1:1 Y2H experiments to further validate the interaction in yeast cells.

### 4.13 BiFC Assay

To perform BiFC assays, the full‐length coding sequences of *FsP4H1*, Δ*SpFsP4H1*, *ScDPb* and *NbDPb* were amplified and inserted between the XbaI‐Bam HI sites of pS1301‐nYFP (C‐terminal MYC tag) or pS1301‐cYFP (C‐terminal HA tag), generating the corresponding fusion constructs (Yuan et al. 2010). Each recombinant plasmid was then introduced into *A. tumefaciens* GV3101 and infiltrated into fully expanded leaves of 4‐ to 5‐week‐old *N*. *benthamiana* plants using a needleless 1 mL syringe. YFP fluorescence in the leaf epidermis was observed using an Olympus FV3000 confocal microscope at 60 h post‐infiltration. All primers used for cloning are listed in Table S1.

### 4.14 Co‐Immunoprecipitation and Western Blotting

To perform coIP assays, the full‐length of *FsP4H1* and *GFP* were amplified and inserted into the ClaI‐NotI sites of the potato virus X (PVX) binary vector pGR107 with a C‐terminal 3×HA tag (FsP4H1‐HA), *ScDPb* and *NbDPb* were amplified and inserted into the KpnI‐BamHI sites of pCambia1300‐FLAG vector with a C‐terminal 3×FLAG (ScDPb/NbDPb‐FLAG), and then each recombinant plasmid was introduced into *A. tumefaciens* GV3101. CoIP was assayed as described previously (Zhu et al. 2023). In brief, the infiltrated leaves of *N. benthamiana* were harvested at 48 h after agroinfiltration, and immediately powdered equally and mixed in extraction buffer (P0013F, Beyotime) at 4°C for 30 min, and then the suspension was centrifuged and filtered to collect the supernatant. Anti‐FLAG magnetic beads (M8823, Millipore) were added to the supernatant and the mixtures were incubated at room temperature for 1 h. After washing three times, the protein‐enriched beads were subjected to western blotting. Anti‐HA (M20013, Abmart) and anti‐FLAG (F1804, Sigma) were used as the primary antibodies and horseradish peroxidase‐conjugated goat anti‐mouse IgG (AS003, Abclonal) was used as the secondary antibody. Signals were developed using chemiluminescence substrate (RM00020P, Abclonal), then imaged using Amersham Imager 680 (GE Healthcare). All primers used for cloning are listed in Table S1.

### 4.15 Transient Expression in *N. benthamiana* to Determine DPb Protein Stability

The empty pGR107 vector, FsP4H1‐HA and ScDPb/NbDPb‐FLAG constructs described above (see ‘Co‐immunoprecipitation and western blotting’) also were used in this experiment. The constructs of empty pGR107 vector, FsP4H1‐HA were adjusted to various concentrations (OD_600_ = 0.2, 0.4 and 0.6), and ScDPb or NbDPb‐FLAG were adjusted to OD_600_ = 0.6. Then, the constructs were added in equal volumes according to the following combinations. (i) FsP4H1‐HA (OD_600_ = 0.2), empty pGR107 vector (OD_600_ = 0.6) and DPb‐FLAG (OD_600_ = 0.6); (ii) FsP4H1‐HA (OD_600_ = 0.4), empty pGR107 vector (OD_600_ = 0.4) and DPb ‐FLAG (OD_600_ = 0.6); (iii) FsP4H1‐HA (OD_600_ = 0.6), pGR107 vector (OD_600_ = 0.2) and DPb‐FLAG (OD_600_ = 0.6). Equal volumes of the prepared constructs were coinfiltrated into *N. benthamiana* leaves to ensure comparability. For the MG132 treatment, *N. benthamiana* leaves infiltrated with (ii) were treated with 100 μm MG132 for 12 h in the dark. About 100 mg of infiltrated leaf tissues was ground into powder in liquid nitrogen and homogenized in 200 μL of extraction buffer (50 mm Tris–HCl, pH 7.5, 150 mm NaCl, 0.5% [v/v] Triton X‐100, 5% glycerol, 1 mm dithiothreitol, 1% protease inhibitor cocktail). Next, 20 μL of the total protein samples was subjected to SDS‐PAGE and immunoblotted with anti‐HA or anti‐FLAG antibody to determine protein levels.

### 4.16 Dual‐Luciferase Reporter Assay

To evaluate the transcriptional impact of ScDPb and NbDPb on their corresponding promoters, a dual‐luciferase reporter assay was performed in *N. benthamiana* via agroinfiltration with *A. tumefaciens* GV3101, following the protocol described previously (Deng et al. 2017). The coding sequences of *FsP4H1*, *ScDPb* and *NbDPb* were amplified and inserted into the HindIII and BamHI sites of the vector pGreenII 62‐SK to obtain the effectors. Promoter fragments of the target genes were amplified and cloned into pGreenII‐0800 to generate the corresponding reporter constructs. The empty pGreenII‐62‐SK vector served as the negative control. Reporter and effector plasmids were co‐introduced into 4‐ to 5‐week‐old *N. benthamiana* leaves by agroinfiltration with *A. tumefaciens* GV3101.

To assess transcriptional stability, the 35S‐driven cassettes containing the CaMV 35S promoter and the full‐length coding sequences of *ScDPb* or *NbDPb* were amplified and cloned into pGreenII‐0800 as a reporter. These reporter plasmids were co‐transformed with the effector construct *FsP4H1*–62‐SK into *A. tumefaciens* GV3101 and pairwise co‐infiltrated into the leaves of 4‐ to 5‐week‐old *N. benthamiana*. Proteasome inhibitor MG132 was infiltrated at 36 h post‐agroinfiltration into *N. benthamiana* leaves co‐expressing FsP4H1 with ScDPb or NbDPb.

## Author Contributions

**Qianqian Ren:** writing – original draft, data curation, investigation, formal analysis. **Muqing Zhang:** project administration, writing – review and editing. **Wei Yao:** writing – review and editing. **Qin Hu:** conceptualization, funding acquisition, formal analysis, supervision, project administration, writing – review and editing. **Shichao Wang:** data curation, formal analysis, investigation. **Chunping Cen:** data curation, formal analysis. **Huifang Li:** data curation, formal analysis. **Yixue Bao:** software, project administration.

## Funding

This work was supported by National Natural Science Foundation of China, 32460070. Science and Technology Major Project of Guangxi, Gui Ke AA24206004. Natural Science Foundation of Guangxi Zhuang Autonomous Region, 2025GXNSFAA069259. State Key Laboratory for Conservation and Utilization of Subtropical Agro‐Bioresources, SKLCUSA‐a05. Ba‐Gui Youth Talent Support Program of Guangxi.

## Conflicts of Interest

The authors declare no conflicts of interest.

## Supporting information


**Figure S1:** Generation of *FsP4H1* knockout and complementation strains in *Fusarium sacchari*.

**Figure S2:** mpp70279‐sup‐0002‐FigureS2.docx. *FsP4H1* from *Fusarium sacchari* was not required for conidia morphology and conidia production.

**Figure S3:** Nuclear localization of FsP4H1 is required for suppression of host immunity.

**Figure S4:** Subcellular localization analysis of ScDPb and NbDPb in *Nicotiana benthamiana* leaves.

**Figure S5:** Schematic representation of the LUC reporter and effector constructs used in transient assays.

**Table S1:** Primer list.

## Data Availability

Sequence data used in this study have been deposited under the following accession numbers. GenBank: *NbPDF1.2*, KP017278.1; *NbPR1*, OQ675540.1; *NbPR2*, XM_019376146.1; *NbHSR203J*, NW_015819496.1; *NbLOX*, KC585517.1; and *NbHIN1*, KU195817. Sol Genomics Network: *NbDPb*, Niben101Scf00801g04021.1; *NbCAT*, Niben101Scf12935g00046.1; China National Center for Bioinformation (project accession GWHEQVP00000000): *ScDPb*, ROC‐Rec‐Chr03A0016840; *ScCAT9*, ROC‐So‐Chr10B0027940; *ScCAT34*, ROC‐Rec‐Chr01A0045830. All other relevant data are available in the article and its Supporting Information.
