# Supplementary material for: The Fusarium sacchari Effector FsP4H1 Targets the Transcription Factor DPb to Disrupt Catalase‐Mediated H2O2 Homeostasis and Suppress Host Immunity
Source: Mol Plant Pathol. 2026 May 19;27(5):e70279. doi: 10.1111/mpp.70279 (PMC13581073; doi:10.1111/mpp.70279)
Supplement: Supplementary file 1 [file MPP-27-e70279-s001.docx]

**Figure S1.** Generation of *FsP4H1* knockout and complementation strains in *Fusarium sacchari.* (a) Schematic of the *FsP4H1* knockout strategy in *F. sacchari.* (b) Schematic of *FsP4H1* complementation in the *ΔFsP4H1* strain. (c) PCR identification of the FsP4H1 knockout mutants (*∆FsP4H1*) using the primer pairs as indicated in (a). (d) Southern blotting for the identification of the *∆FsP4H1* transformants by probe Hyg. (e) PCR identification of the *FsP4H1* complementary mutants (*Com∆FsP4H1*) using the primer pairs as indicated in (b).
