# Supplementary material for: The Fusarium sacchari Effector FsP4H1 Targets the Transcription Factor DPb to Disrupt Catalase‐Mediated H2O2 Homeostasis and Suppress Host Immunity
Source: Mol Plant Pathol. 2026 May 19;27(5):e70279. doi: 10.1111/mpp.70279 (PMC13581073; doi:10.1111/mpp.70279)
Supplement: Supplementary file 3 [file MPP-27-e70279-s003.docx]

**Figure S3.** Nuclear localization of FsP4H1 is required for suppression of host immunity. (a) Subcellular localization of NES-FsP4H1-GFP and NLS-FsP4H1-GFP in representative leaves imaged at 60 h post-infiltration. (b) Lesion diameters on *N. benthamiana* leaves expressing the indicated constructs after *B. cinerea* inoculation; UV-illuminated images at 2 dpi. (c) Lesion areas were quantified at 2 days post *B. cinerea* inoculation in *N. benthamiana* leaves transiently expressing the indicated constructs. Data are means ± SD, n = 6. Different letters denote significant differences (One-way ANOVA; P < 0.05). Scale bar = 20μm.
