# Supplementary material for: The Fusarium sacchari Effector FsP4H1 Targets the Transcription Factor DPb to Disrupt Catalase‐Mediated H2O2 Homeostasis and Suppress Host Immunity
Source: Mol Plant Pathol. 2026 May 19;27(5):e70279. doi: 10.1111/mpp.70279 (PMC13581073; doi:10.1111/mpp.70279)
Supplement: Supplementary file 4 [file MPP-27-e70279-s004.docx]

**Figure S2.** *FsP4H1* from *F. sacchari* was not required for conidia morphology and conidia production. (a)-(b) Statistical analysis of the conidia production of *ΔFsP4H1, ComΔFsP4H1,* and wild-type strains grown on the medium with PDA, pectin, cellulose, or sucrose as a carbon source. Data are means ± SD, n = 6. Bar: 10 μm. (c) The conidia morphology of *ΔFsP4H1, ComΔFsP4H1,* and wild-type strains grown on medium with PDA, pectin, cellulose, or sucrose as a carbon source for 7 days.
