# Supplementary material for: The Fusarium sacchari Effector FsP4H1 Targets the Transcription Factor DPb to Disrupt Catalase‐Mediated H2O2 Homeostasis and Suppress Host Immunity
Source: Mol Plant Pathol. 2026 May 19;27(5):e70279. doi: 10.1111/mpp.70279 (PMC13581073; doi:10.1111/mpp.70279)
Supplement: Supplementary file 5 [file MPP-27-e70279-s005.docx]

**Figure S4.** Subcellular localization analysis of ScDPb and NbDPb in *N. benthamiana* leaves. Constructs of ScDPb and NbDPb were transiently overexpressed in *N. benthamiana* leaves. Bar: 20 μm.
