# Supplementary material for: The Fusarium sacchari Effector FsP4H1 Targets the Transcription Factor DPb to Disrupt Catalase‐Mediated H2O2 Homeostasis and Suppress Host Immunity
Source: Mol Plant Pathol. 2026 May 19;27(5):e70279. doi: 10.1111/mpp.70279 (PMC13581073; doi:10.1111/mpp.70279)
Supplement: Supplementary file 6 [file MPP-27-e70279-s006.docx]

Table S1 Primer list

| Primer name | Sequences (5'-3') | Note |
| --- | --- | --- |
| SpFsP4H1-pSUC2-F | GGAATTTTAATTAAGAATTCTTTCAATACGTGATTGGGCT | Yeast Signal Sequence Trap System |
| SpFsP4H1-pSUC2-R | CTATAGGGAGAACCTCGAG TGAGAACACTAGAATAATAG | Yeast Signal Sequence Trap System |
| FsP4H1-GFP-F | GTCCGGACTCAGATCTCGAGATGTTTCAATACGTGATTGG | Subcellular Localization |
| FsP4H1-GFP-R | GACTGCAGAATTCGAAGCTTGATTCGTCCAAAACTCCAGA | Subcellular Localization |
| ΔSpFsP4H1-GFP-F | GTCCGGACTCAGATCTCGAGATGAACCCCATCAGTGACTT | Subcellular Localization |
| ScDPb-GFP-F | GTCCGGACTCAGATCTCGAG ATGGCGCCCCCCTGCGGCGA | Subcellular Localization |
| ScDPb-GFP-R | GACTGCAGAATTCGAAGCTTGCCCGCTCTTTTTATGCTAT | Subcellular Localization |
| NbDPb-GFP-F | GTCCGGACTCAGATCTCGAGATGGTGAATAATTTGGATGA | Subcellular Localization |
| NbDPb-GFP-R | GACTGCAGAATTCGAAGCTTATGCTCATGCTTGATGCGGG | Subcellular Localization |
| NES-FsP4H1-F | GCTTCGTTTTGAATCGTCGAATGTTTCAATACGTGATTGG | Subcellular Localization |
| NES-FsP4H1-R | TCGCCCTTGCTCACCATCAGGATTCGTCCAAAACTCCAGA | Subcellular Localization |
| NES-ΔSPFsP4H1-F | GCTTCGTTTTGAATCGTCGAATGAACCCCATCAGTGACTT | Subcellular Localization |
| NLS-FsP4H1-F | CCCGATCCGCTGCAGTCGACATGTTTCAATACGTGATTGG | Subcellular Localization |
| NLS-FsP4H1-R | CCCTTGCTCACCATCACTAGGATTCGTCCAAAACTCCAGA | Subcellular Localization |
| FsP4H1-pGKO-up-F | AGCTCGCTGAGGGTTTAACTCGGGTGCTATTTCGCTAC | Transformations for Gene Deletion and Complementation |
| FsP4H1-pGKO-up-R | GGCCCGCTGAGGACTTAAGGCTGAGGGCAACTAATAAG | Transformations for Gene Deletion and Complementation |
| Fs00176-PGKO-down-F | ACTAGTGCTGAGGCATTAAGGGTGTTTGTGTTCAGTAAT | Transformations for Gene Deletion and Complementation |
| Fs00176-PGKO-down-R | AAGCTTGCTGAGGTCTTAAAGGACTACATGCACGGCGATC | Transformations for Gene Deletion and Complementation |
| FsP4H1-F | ATGTTTCAATACGTGATTGG | Transformations for Gene Deletion and Complementation |
| FsP4H1-R | TTAGATTCGTCCAAAACTCC | Transformations for Gene Deletion and Complementation |
| HPT-F | ACACTACATGGCGTGATTTCAT | Transformations for Gene Deletion and Complementation; Hyg probe clone |
| HPT-R | TCCACTATCGGCGAGTACTTCT | Transformations for Gene Deletion and Complementation;  Hyg probe clone |
| HPT-up-R | TGAATCTGAAATAAAGGGAGGA | Transformations for Gene Deletion and Complementation |
| HPT-down-F | GACTGAGGAATCCGCTCTTGG | Transformations for Gene Deletion and Complementation |
| FsP4H1-up-F | CTCGGGTGCTATTTCGCTAC | Transformations for Gene Deletion and Complementation |
| FsP4H1-down-R | AGGACTACATGCACGGCGAT | Transformations for Gene Deletion and Complementation |
| FsP4H1-pDHtsk-F | GGTCGACGGTATCGATAAGCTT ATGTTTCAATACGTGATTGG | Transformations for Gene Deletion and Complementation |
| FsP4H1-pDHtsk-R | GAGGGTTAATTGCGCGGATCCTTAGATTCGTCCAAAACTCC | Transformations for Gene Deletion and Complementation |
| G418-F | CTCTGATGCCGCCGTGTTCC | Transformations for Gene Deletion and Complementation |
| G418-R | GCGGCGATACCGTAAAGCAC | Transformations for Gene Deletion and Complementation |
| FsP4H1-PVX-F | GCACCAGCTAGCATCGATATGTTTCAATACGTGATT | *Agrobacterium*-mediated Transient Expression in *N. benthamiana* |
| FsP4H1-PVX-R | GTATGGGTACGCGGCCGCGATTCGTCCAAAACTCCA | *Agrobacterium*-mediated Transient Expression in N. benthamiana |
| ∆SpFsP4H1-PVX-F | GCACCAGCTAGCATCGATATGAACCCCATCAGTGACTT | *Agrobacterium*-mediated Transient Expression in *N. benthamiana* |
| ScDPb-PVX-F | GCACCAGCTAGCATCGAT ATGGCGCCCCCCTGCGGCGA | Agrobacterium-mediated Transient Expression in *N. benthamiana* |
| ScDPb-PVX-R | GTATGGGTACGCGGCCGCGCCCGCTCTTTTTATGCTAT | *Agrobacterium*-mediated Transient Expression in *N. benthamiana* |
| NbDPb-PVX-F | GCACCAGCTAGCATCGAT ATGGTGAATAATTTGGATGA | *Agrobacterium*-mediated Transient Expression in *N. benthamiana* |
| NbDPb-PVX-R | GTATGGGTACGCGGCCGCATGCTCATGCTTGATGCGGG | *Agrobacterium*-mediated Transient Expression in *N. benthamiana* |
| ScCAT34-PVX-F | GCACCAGCTAGCATCGATATGGACCCGTACAAGGTACG | *Agrobacterium*-mediated Transient Expression in *N. benthamiana* |
| ScCAT34-PVX-R | GTATGGGTACGCGGCCGCCGTCCAGCGGGTCGAAGTCG | *Agrobacterium*-mediated Transient Expression in *N. benthamiana* |
| ScCAT9-PVX-F | GCACCAGCTAGCATCGATATGGATCCGTACAAGCACCG | *Agrobacterium*-mediated Transient Expression in *N. benthamiana* |
| ScCAT9-PVX-R | GTATGGGTACGCGGCCGCCATGTTTGGCTTCAGGTTCA | *Agrobacterium*-mediated Transient Expression in *N. benthamiana* |
| NbCAT-PVX-F | GCACCAGCTAGCATCGATATGGTGAATAATTTGGATGA | *Agrobacterium*-mediated Transient Expression in *N. benthamiana* |
| NbCAT-PVX-R | GTATGGGTACGCGGCCGCATGCTCATGCTTGATGCGGG | *Agrobacterium*-mediated Transient Expression in *N. benthamiana* |
| ΔSPFsP4H1-BD-F | TGGCCATGGAGGCCGAATTCATGAACCCCATCAGTGACTT | yeast two-hybrid |
| ΔSPFsP4H1-BD-R | TGCAGGTCGACGGATCCGGAGTTTTGGACGAATCTAA | yeast two-hybrid |
| ScDPb-AD-F | TGGAGGCCAGTGAATTCATGGCGCCCCCCTGCGGCGA | yeast two-hybrid |
| ScDPb-AD-R | TCGAGCTCGATGGATCCCTAGCCCGCTCTTTTTATGC | yeast two-hybrid |
| NbDPb-AD-F | TGGAGGCCAGTGAATTCATGGTGAATAATTTGGATGA | yeast two-hybrid |
| NbDPb-AD-R | TCGAGCTCGATGGATCCCTAATGCTCATGCTTGATGC | yeast two-hybrid |
| ScCAT9-pHis2-F | ACTCACTATAGGGCGGAATTCGAGAGCAATGGATATCGGAG | Yeast one-hybrid |
| ScCAT9-pHis2-R | ATTCGCGAACGCGTGAGCTCGGCTGCCGGTGGGATCGCGT | Yeast one-hybrid |
| ScCAT34-pHis2-F | ACTCACTATAGGGCGGATTCCTCCTCGTCGCGGTGCATG | Yeast one-hybrid |
| ScCAT34-pHis2-R | ATTCGCGAACGCGTGAGCTCCCAAGACGTGGCCCGAGGAC | Yeast one-hybrid |
| NbCAT-pHis2-F | ACTCACTATAGGGCGAATTCACACGTTTCAAGATCCTTTT | Yeast one-hybrid |
| NbCAT-pHis2-R | ATTCGCGAACGCGTGAGCTCGGCGAGAAAATATGAGCGAG | Yeast one-hybrid |
| ΔSPFsP4H1-nYFP-F | GGCGCGCCACTAGTGGATCCATGAACCCCATCAGTGACTT | BiFC |
| ΔSPFsP4H1-nYFP-R | TCCATCCCGGGAGCGGTACCGATTCGTCCAAAACTCCAGA | BiFC |
| FsP4H1-nYFP-F | GGCGCGCCACTAGTGGATCCATGTTTCAATACGTGATTGG | BiFC |
| ScDPb-cYFP-F | GGCGCGCCACTAGTGGATCCATGGCGCCCCCCTGCGGCGA | BiFC |
| ScDPb-cYFP-R | TCCATCCCGGGAGCGGTACCGCCCGCTCTTTTTATGCTAT | BiFC |
| NbDPb-cYFP-F | GGCGCGCCACTAGTGGATCCATGGTGAATAATTTGGATGA | BiFC |
| NbDPb-cYFP-R | TCCATCCCGGGAGCGGTACCATGCTCATGCTTGATGCGGG | BiFC |
| FsP4H1-62SK-F | CGCTCTAGAACTAGTGGATCCATGTTTCAATACGTGATTGG | LUC |
| FsP4H1-62SK-R | TCGACGGTATCGATAAGCTTTTAGATTCGTCCAAAACTCC | LUC |
| 35S-0800-F | TCGACGGTATCGATAAGCTTTGAGACTTTTCAACAAAGGG | LUC |
| ScDPb-LUC-R | GCTCTAGAACTAGTGGATCCGCCCGCTCTTTTTATGCTAT | LUC |
| NbDPb-LUC-R | GCTCTAGAACTAGTGGATCCATGCTCATGCTTGATGCGGG | LUC |
| ScDPb-62SK-F | CGCTCTAGAACTAGTGGATCCATGGCGCCCCCCTGCGGCGA | LUC |
| ScDPb-62SK-R | TCGACGGTATCGATAAGCTTCTAGCCCGCTCTTTTTATGC | LUC |
| NbDPb-62SK-F | CGCTCTAGAACTAGTGGATCCATGGTGAATAATTTGGATGA | LUC |
| NbDPb-62SK-R | TCGACGGTATCGATAAGCTTCTAATGCTCATGCTTGATGC | LUC |
| ProScCAT34-0800-F | TCGACGGTATCGATAAGCTTCCTCCTCGTCGCGGTGCATG | LUC |
| ProScCAT34-0800-R | GCTCTAGAACTAGTGGATCCAGACGTGGCCCGAGGACGTG | LUC |
| ProScCAT9-0800-F | TCGACGGTATCGATAAGCTTGAGAGCAATGGATATCGGAG | LUC |
| ProScCAT9-0800-R | GCTCTAGAACTAGTGGATCCTGCCGGTGGGATCGCGTCGA | LUC |
| ProNbCAT-0800-F | TCGACGGTATCGATAAGCTTACACGTTTCAAGATCCTTTT | LUC |
| ProNbCAT-0800-R | GCTCTAGAACTAGTGGATCCGAGAAAATATGAGCGAGAAG | LUC |
| Q-FsP4H1-F | GCACCGATTTTCCCCTCAT | RT-qPCR |
| Q-FsP4H1-R | CGCCGCAAAGTTCTCCCAA | RT-qPCR |
| NbEF1α-F | GGTTAAGATGATGCCGACCAAG | RT-qPCR |
| NbEF1α-R | CGCCAGTTGGGTCCTTCTTG | RT-qPCR |
| Fv_Actin-F | GAGAACGAGCGTGTCTTGATTGAGCC | RT-qPCR |
| Fv_Actin-R | TTCCICCGCAGAATGAAGAAGGACTC | RT-qPCR |
| NbPR1-F | CCGCCTTCCCTCAACTCAAC | RT-qPCR |
| NbPR1-R | GCACAACCAAGACGTACTGAG | RT-qPCR |
| NbPR2-F | AGGTGTTTGCTATGGAATGC | RT-qPCR |
| NbPR2-R | TCTGTACCCACCATCTTGC | RT-qPCR |
| NbPDF1.2-F | AGGGACCATGCTCAAGAGA | RT-qPCR |
| NbPDF1.2-R | AGTACAGAAACAACGGCGG | RT-qPCR |
| NbLOX-F | AAAACCTATGCCTCAAGAAC | RT-qPCR |
| NbLOX-R | ACTGCTGCATAGGCTTTGG | RT-qPCR |
| NbHSR203-F | GGCAGTGGAGGAGCTTAAAT | RT-qPCR |
| NbHSR203-R | GCTATGTCCCACTCCATTGTTA | RT-qPCR |
| NbH1N1-F | ATCCTCGGAGTGATTGCATTAG | RT-qPCR |
| NbH1N1-R | TGTTGTTTGTGGTGGACAAATC | RT-qPCR |
